# Availability of adequately iodized salt at household level in Dessie and Combolcha Towns, South Wollo, Ethiopia

**DOI:** 10.1186/s12889-018-6066-5

**Published:** 2018-10-03

**Authors:** Tefera Chane Mekonnen, Sisay Eshete, Yitbarek Wasihun, Mastewal Arefaynie, Nigus Cherie

**Affiliations:** 1 Human Nutrition Unit, Department of Public Health, Wollo University, Dessie, Ethiopia; 2 Department Public Health, Wollo University, Dessie, Ethiopia; 3 Reproductive Health Unit, Department of Public Health, College of Medicine and Health Sciences, Wollo University, Dessie, Ethiopia

**Keywords:** Adequacy, Iodized salt, Household level, Dessie, Combolcha, Ethiopia

## Abstract

**Background:**

Iodine deficiency disorder is the leading cause of mental retardation and poor economic performance in developing countries. Worldwide, universal salt iodization has been implemented to eliminate iodine deficiency. However, the adequacy of iodine in salts needs close monitoring to meet its intended goal and this study was aimed at investigating the adequacy of iodine in dietary salt at household level in Dessie and Combolcha Towns.

**Methods:**

A community-based cross-sectional study was employed at household level in Dessie and Combolcha towns from January to February, 2017. Data were collected from 753 households using systematic sampling technique. The adequacy of iodine in salt was analyzed using rapid testing kit. Socio-demographic and economic, dietary sources, labeling, packaging, storage and cooking methods of household’s characteristics were collected via questionnaire developed using open data kit tool and STATA version 12 was used for further statistical analysis. Ordinal Logistic regression was performed to assess associations between explanatory variables and the response variable.

**Results:**

Nearly one-thrid (31.2%) of the households used inadequate iodized salt, which was below the World Health Organization recommendation level (≥15 ppm at the household level). Most of the respondents from Combolcha town (64.6%) were affected by inadequate use of iodized salt as compared to Dessie Town residents (22.2%). Being Dessie resident (OR = 2.53; 95% CI: 1.31–4.90), households with better socioeconomic status (OR = *2.54; 95% CI:1.10–5.87)*, site of labeling and packing (salt from open market (OR = *0.10; 95% CI: 0.04–0.23)* and no exposure to sunlight (OR = *2.54; 95% CI:1.31–4.91)* were the predictors of adequacy of iodized salt at household level.

**Conclusions:**

Availability of adequately iodized salt at the household level in the study area was low. There should be regular quality control and regulatory enforcement of salt iodization at production, labeling and packaging sites of small scale industries and at household level.

## Background

Iodine is an essential element for normal function of the thyroid gland. Its deficiency is the most common cause of preventable mental retardation and brain damage in the world [1–3]. Iodine deficiency disorders (IDD) is manifested in multiple adverse health consequences like decreased child survival rates, goitre, abortion, stillbirth, malformation and overall impaired growth and development [1, 4, 5] and remained major public health challenge and an obstacle to economic development worldwide [6, 7]. Iodine deficiency remains a public-health problem in many low-income countries, and more than a billion individuals consumed an insufficient dietary iodine [8]. Reports revealed that 38 million children born every year are at risk of brain damage because of iodine deficiency [9].

In Ethiopia, around 28 million people suffer from goitre, and more than 35 million people are at risk of iodine deficiency. More importantly, 50,000 perinatal deaths are related to iodine deficiency each year in Ethiopia. The education potential of the nation is unattained as iodine deficiency may cause an intelligence quotient reduction of 13.5 points [9]. The problem is both a threat to the productivity of the workforce and a cause of cretinism and mental retardation which lead to an enormous loss of gross domestic product [10].

WHO recommends a daily intake of 90 μg of iodine for preschool children, 120 μg for schoolchildren, 150 μg for adolescents and adults, and 200 μg for pregnant and lactating women [11]. WHO recommended Universal Salt Iodization (USI) for elimination of IDDs [11] a daily intake of salt by all members of the population should provide the required amount of iodine [12].

Salt iodine testing is an important process indicator for monitoring progress towards Universal Salt Iodization (USI) [13]. WHO/UNICEF/ICCIDD recommends that the salt iodine content at the packaging level must be 50–60 ppm and 20–30 ppm at the retail shops, to achieve at least 15 ppm in the household dietary consumption [2]. The salt iodine content is estimated by rapid test kit(RTK) methods which measures of the iodine content [11].

Iodization of salt is currently carried out following the USI initiative [14, 15]. The concentration of iodine from iodized salt can be influenced by the variability of iodine during iodization process, packing with bags and instability of iodine in the salt. Moreover, moisture content of salt, humidity, light, heat, impurities in salt, alkalinity or acidity, and the form in which the iodine is present, affect iodine stability in salt [16].

Recent monitoring of the iodine indicated that 62% of households used adequately iodized salt containing at least 15 ppm iodine in Laelay Maychew District, Northern Ethiopia and South Africa [17–19], considerably below the international goal of 90% coverage [11].

According to the Ethiopia Demography and Health Survey 2016, 89% of the Ethiopian population uses iodized salt [20]. Measuring iodine concentrations and documentation of losses at the consumers’ level are essential elements of a programme to eliminate iodine deficiencies. Additionally, it is important to monitor what percentage of the population remains at risk of insufficient intake of iodine. Therefore, this study aimed to investigate the adequacy of iodine from dietary salt at household level.

## Methods

### Study design and area

A Community-based, cross-sectional study design was carried out from January 18–30, 2017 in Dessie and Combolcha towns. Dessie is the capital city of South Wollo administrative Zone, and is one of the three metropolitan towns in the Amhara Regional State. It is located in the Northern part of Ethiopia about 401 Kms from the capital city, Addis Ababa to the North East, and about 480 Kms away from the capital city of the Amhara Regional State, Bahir-Dar, to the east. According to the 2013–2014 years, the total population in the town is 195,661 (male 94,285, female 101,376).

Combolcha town is also 374 kms far away from Addis Ababa and it is one of the industrial zones in the nation. Administratively Combolcha is structured in 11 Administrative Units (called Kebeles in Amharic). The town has relatively hot climatic condtion as compared to Dessie. According to 2015/16, the total households are 30, 631, in which 23, 102 (75.4%) households are living in urban areas and 7, 529 households are residing in rural areas. Housewives or household heads that had been living for at least 6 months and above in the randomly selected Kebeles were included in the study. Severely ill individuals and those who cannot prepare foods in their home were excluded from the study.

Sample size was determined using single population proportion formula by considering the prevalence of insufficient iodine intake at household level in Tigray a nearby region in Ethiopia as 63% [20], 95% confidence level based on standard normal distribution, 5% as degree of precision and 1.5 as design effect. Assuming 5% for non-respondent, the sample size was 524 households.

Multistage sampling technique was used to select target respondents. Dessie city administration has ten administrative units (Kebeles) and Combolcha town has five administrative units (Kebeles). Then three Kebeles from Dessie town and one Kebele from Combolcha town were selected randomly. The sample was allocated proportionally to the size of households in the two areas. The allocated sample for Dessie town was reallocated to the three Kebeles based on the total households resided. The total households in the selected Kebeles of the two Towns were divided to the allocated sample sizes to get the sampling interval and the study subjects were systematically drawn.

### Data measurement and quality control

All required data were assembled using a questionnaire organized from EDHS, UNICEF procedure protocol and from other studies [11, 20, 21] via Open Data Kit (ODK) tool. The questionnaire was compiled from validated pre-established sources and was modified contextually as per the objectives of the study [2, 10, 19–22]. Six data collectors who had Bachelor of Science (BSc) in medical laboratory technicians with previous data collection experiences, were recruited. Additionally, two Master of Public Health (MPH) professionals with previous experiences in handling survey field work were selected as supervisors.

Before the commencement of actual survey, pre-test was conducted on 5% of the sample in areas found other than the study site. Detailed investigation was carried out to shuffle the tool in order to address the desired study objective. A 3 days intensive training was given for data collectors and supervisors on how to record, compile and complete the questionnaire. The RTK was done under close supervision and if there is any suspicion the test was repeated.

Determination of iodine content from dietary salt by using RTK, socio-demographic, economic variables, sources of iodized salt, knowledge about iodine and its effect, common food sources, container/shelf of iodized salt, timing of addition during cooking and other associated factors were obtained by interview using smart phones.

One medium sized tea spoon of iodized salt was taken from each households. The semi-quantitative estimation of the salt iodine by using a RTK is based on the reaction between starch and iodine to form starch-iodine complex. This test solution contains an acidic buffer and a reducing agent, which convert potassium iodate (KIO_3_) to elemental iodine (I_2_) [11].

The white cup found from the test kit was filled with salt and spread the salt surface flat first. Secondly, two drops of the test solution on the surface of the salt were added and the colour of the test sample is compared with the standard colour chart (< 15 ppm or > 15 ppm) [13, 16], within 1 min and iodine content was determined. If the colour of test sample did not appear after 1 min, three to five drops of the recheck solution were added on a fresh sample, followed by two drops of test solution were added on the same spot and then the colour was compared with the colour chart to record the content (recorded as “adequate” for blue colour, as “Low” for gray colour and as “nil/zero” for white colour).

### Data management and analysis

The data collected by ODK software were uploaded to cloud server of the user account. Once the data had been collected and stored, it was downloaded in the form of comma delimited file type. Then, it was finally transported to STATA version 12 for analysis. The data were explored for missing data, distribution of outcome variable, and test of parallel lines and model fitness information.

The association between the adequacy of iodized salt and related factors was performed through ordinal logistic regression. The main effect was specified for the location of the proportional odds model, test of parallel lines checked (*p*-value = 0.34), Goodness of fit statistics was checked (*P*-value = 0.67) and the Link function was Logit during the analysis. Candidate variables with *p*-value < 0.2 moved to the final model to identify predictor variables and finally, a *p*-value of < 0.05 (two-tailed) was used to declare statistically significant variables.

Socio-economic status of the households was measured using assets by Principal Component analysis. After check the assumption of sample adequacy, communality and presences of complex struction, the score was ranked from the first (poorest) to fifth (richest) quintile (constituting 20% of distribution in each category).

## Results

### Socio-demographic and economic characteristics

A total of 500 households in Dessie and Combolcha Towns gave complete responses on adequacy of iodized salt. About three-fourth of the households (77.4%) were from Dessie Town residents and the remaining were from Combolcha (Table 1). The mean age of respondents was 33 years (±10.5 standard deviation). Among all respondents, about 91% of the household members who involved in the preparation of food were women. Of all study participants, about 78% respondents were married.

**Table 1:** Showed Socio-Demographic characteristics of households from Dessie and Combolcha Town residents, June 2017

Variables (*n* = 500)	Frequency	Percent
Residents		
Dessie	387	77.4
Combolcha	113	22.6
Sex of respondents		
Female	475	95.0
Male	25	5.0
Household involved in food preparation		
Wife	457	91.0
Husbands	43	9.0
Age of respondents(in years)		
≤ 25 years	148	29.6
26–34 years	142	28.4
≥ 35 years	210	42.0
Respondents educational Status		
Formal	338	67.6
Informal	162	32.4
Religion		
Muslim	267	53.4
Orthodox	217	43.4
Others	16	3.2
Marital status of the respondents		
Married	391	78.2
Single	109	21.8
Respondents occupational status		
Housewife	339	67.8
Farmer	79	15.8
Private employee	46	9.2
Government employee	36	7.2
Households’ wealth Status		
First quintile	106	21.2
Second quintile	78	15.6
Third quintile	108	21.6
Fourth quintile	115	23.0
Fifth quintile	93	18.6
Role of respondents regarding with food		
Preparation only	106	21.2
Preparation and recruitments	394	78.8

### Respondents’ information regarding iodine

In this study, about one-fifth of the participants had not heard about iodine. Among study subjects who heard about iodine, more than half of them obtained information from mass media (television and radio) about iodine. Three fourth of the respondents believed that deficiency of iodine brings development of goiter only. More than 80% of respondents replied that iodine deficiency disorder is preventable. Regarding the dietary sources of iodine, 25% of the study participants knew the food sources that contain iodine (Table 2).

**Table 2:** Participants’ information regarding iodine health importance and its deficiency in Dessie and Combolcha residents, June 2017

Items	Frequency	Percent
Have ever heard about Iodine?		
Yes	403	80.6
No	97	19.4
Sources of information for iodine		
From friends and relatives only	22	5.5
From Health Extension Workers only	140	34.7
From Television only	142	35.2
From Health extension workers and Television	99	24.6
Respondents response on the effect of IDD (*n* = 403)		
Goiter only	303	75.2
Goiter, abortion, mental retardation and cretinism	77	19.2
cancer	1	0.2
anemia	1	0.2
Did not know	21	5.2
Do you know Goiter?		
Yes	461	92.2
No	39	7.8
Causes of goiter		
Iodine deficiency	364	72.8
Hereditary	42	8.4
Evil	21	4.0
Infection	70	14.0
Poisoning	24	4.8
Is IDD preventable?		
Yes	417	83.4
No	83	16.6
Know dietary sources of iodine		
Yes	126	25.2
No	358	71.6
Not sure	16	3.2
Do you buy iodized salt?		
Yes	394	78.8
No	106	21.2
Site of production (package place)		
Afdera	136	27.2
Mesobo	56	11.2
Open	125	25.0
Shewit	183	36.6
Reason for buying iodide salt		
Good for health only	347 (69.4)	69.4
Advice from health professional	31 (6.2)	6.2
Has no option	32 (6.4)	6.4
Did not buy	90 (18.0)	18.0
Types of pack		
Packed	395 (79.0)	79.0
Unpacked	105 (21.0)	21.0
Do you know the benefits of iodized salt?		
Yes	392 (78.4)	78.4
No	108 (21.6)	21.6
Benefit of iodized salt (*n* = 392)		
Has good taste and palatability	113 (28.8)	28.8
Prevent goiter and other IDDs	279 (71.2)	71.2
Preference for salt		
Iodized	402 (80.4)	80.4
Non-iodized	32 (6.4)	6.4
No preference	66 (13.2)	13.2

Regarding the place of storage of iodized salt, almost all households placed it in a dry place. The majority of the participants (96.4%) reported that the salt kept under cover, but 26.2% of the respondents exposed for sunlight before storage. Regarding the use of salt, 80.6% of the participants stated that they added the salt at the end of cooking while 15.6% of them added at the middle of cooking.

### Adequacy of iodized salt

**Fig. 1:**
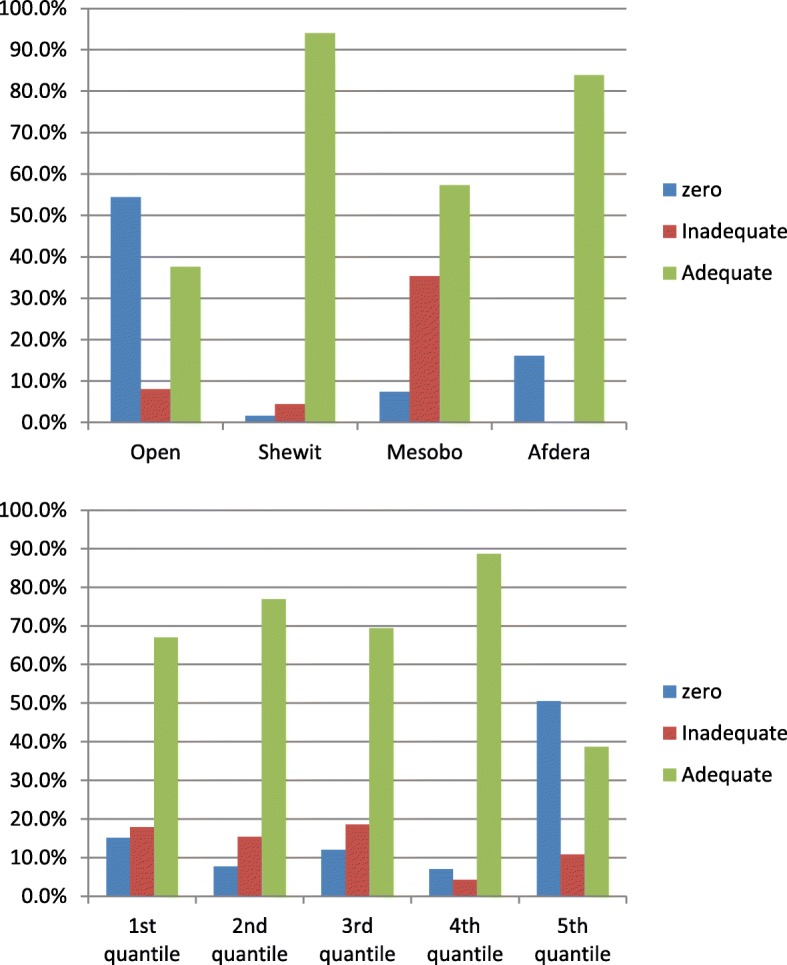
Consumption of iodized salt at household level by the site for packing and labeling of salt in Dessie and Combolcha Town, 2017

**Fig. 2:**
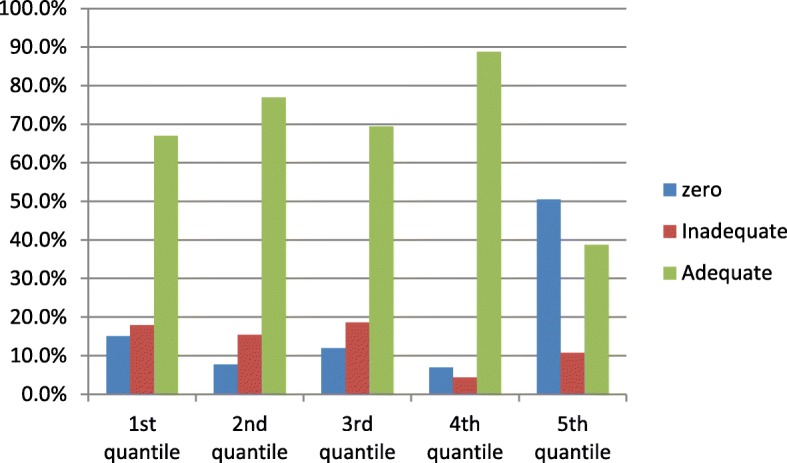
Consumption of iodized salt at household level by their socio-economic statusin Dessie and Combolcha Town, 2017

Household dietary salt was determined by using rapid test kit duringthe data collection period. Among all respondents,18% (95% CI: 14.8–21.2%) of the households used salt without iodine (zero iodine concentration) and 13.2% (95% CI: 10.1–16.1%) of the respondents used salt with inadequately iodized (below 15 ppm) whereas, 68.8% (95% CI: 64.8–72.9%) of the salt sample was adequately iodized. Area specific iodized salt consumption, the report revealed that about 62% of the respondents of Combolcha residents used salt with zero iodine contents while 22.3% of households of Dessie town used inadequately iodized salt. The highest percentage of no iodine content was observed among farmer respondents (21.1%) followed by respondents from housewives (19.4%) and the least was found from government employees (9.6%). In salts bought from open market, 54% was not iodized (Figs. 1 and 2).

### Factors related with adequacy of iodized salt at household level

Among variables entered to ordinal logistic model, area of residents (Dessie vs Combolcha), socio-economic status, site for labeling and packing of iodized salt and exposure of iodized salt for sunlight were found to have significant association with the adequacy of iodized salt at household levels. The odds of iodized salt adequacy among Dessie residents were almost three times higher as compared to the Odds of iodized salt adequacy among Combolcha Residents (OR=2.53; 95% CI: 1.31-4.90). Moreover, The probability of adequacy of iodized salt decreased by 90% if open market salt was used. (OR=*0.10; 95% CI: 0.04-0.23)* (Table 3). However, independent variables like marital status of the respondent, Educational status of the respondent and occupational status of the respondents did not show statistically significant association with adequacy of iodized salt*.*

**Table 3:** Factors related with Iodized salt adequacy at household level among Dessie and Combolcha Town residents in May 2017

Variables	Test result of Iodine by RTK			COR (95% CI)	AOR (95% CI)
	Nil N (%)	Inadequate N (%)	Adequate N (%)		
Place of residence					
Dessie	20	66	301	10.91 (6.68–16.44)	*2.53 (1.31–4.90)*
Combolcha	65	8	40	1	*1*
Marital status of the respondent					
Married	68	46	277	2.07 (0.89–4.85)	0.92 (0.55–1.55)
Single	22	20	67	1	1
Educational status of respondents					
Formal	43	48	243	1.91 (1.29–2.80)	1.79 (0.97–3.28)
Informal	47	18	101	1	1
Occupational status					
Farmer	16	8	55	1.05 (0.63–1.76)	1.16 (0.68–1.97)
Employed	8	15	59	1.32 (0.78–2.27)	1.37 (0.80–2.35)
Housewife	66	43	230	1	1
Socioeconomic status					
First quintile (poorest)	16	19	71	4.18 (2.38–7.31)	1.66 (0.85–3.25)
Second quintile	6	12	60	6.96 (3.60–13.46)	2.05 (0.93–4.51)
Third quintile	13	20	75	4.76 (2.72–8.41)	1.90 (0.96–3.75)
Fourth quintile	8	5	102	15.33 (7.69–30.57)	*2.54 (1.10–5.87)*
Fifth quintile(Richest)	47	10	36	1	*1*
Site for labeling and packing of iodized salt					
Open market	68	10	47	0.08 (0.04–0.19)	*0.10 (0.04–0.23)*
Shewit	3	8	172	3.38 (1.35–8.50)	1.25 (0.92–3.49)
Mesobo	10	48	78	0.36 (0.17–0.77)	*0.18 (0.08–0.43)*
Afdera	8	4	44	1	*1*
Iodized salt exposure for sunlight					
No	18	59	292	9.21 (5.99–14.01)	*2.54 (1.31–4.91)*
Yes	72	7	52		1

## Discussion

The study found that the overall percentage of households who used inadequate iodized salt (< 15 ppm)was 31.2%. The iodized salt inadequacy was more severe in Combolcha residents. The finding of this study revealed that adequacy of iodized salt was much higher than reports from rural community of Maychew (33%) [21], West Ethiopia (8.7%) [22] and Gondar town, Ethiopia (28.9%) [23] and Asella Town, Ethiopia (62.9%) [24]; and similar result was observed from urban area of Sidama Zone, Ethiopia (65%) [25]. Even though there are many variation and strategies across countries, the finding of the study was also higher as compared to findings from other low income contries [13, 26]. In contrast to the above finding, adequacy of iodized salt in the study was lower than findings from EasternNepal (82.6%) [27] and Telangana, India (79%) [3].

The reason for these variations may be due to relatively wide time elapsed between the current study and others. Additionally, controlling of iodine deficiency disorders has been given universal attention with strong monitoring and evaluation of iodine from dietary salt at every levels (at production, transportation and consumption levels). The differences in agro-ecological situations, socio-economic status and perceived knowledge and practices among households may bring the above discrepancies in different areas.

Proportional Odds model with Logit link function was used to identify predictors of iodized salt adequacy at household level. The model identified place of residence, households in the fourth quintile of socio-economic class, packing and labeling site and exposure for sunlight as having statistical significant association with adequacy of iodized salt at consumption level. The salt samples brought from Dessie household residents were found to be more likely adequately iodized as compared to salt samples from Combolcha Town. This report was in line with the WHO recommendation report [2], finding from studies conducted in West Ethiopia [22], Eastern Nepal [27] and Telangana, India [3] indicated that the level of moisture and humidity of areas affect the content of iodine in the salt. Additional reasons may be most of the households of Combolcha Town used open market salt for cooking and they exposed the salt to sunlight.

Households found in higher socio-economic categories showed more significant observed difference in better adequacy of iodized salt use as compared to households found in low socio-economic status. Many studies agreed that iodized salt was not accessible for most of the poor society in the world. Reports from Asella town [24], Sidama Zone [25] and rural India [26] postulated that the better socio-economic status, the more adequately iodized salt used and agreed that packed iodized salt is not affordable for the poor.

Regarding place of iodized salt where it was packed and labeled; households who used open market salt were less likely to use adequately iodized salt. Study conducted by Kumar A., et al. in Eastern Nepal [27] found that there is a significant association between use of open market salt and inadequacy of iodine content in dietary salt. Similarly studies from West Ethiopia [22], Sidama, SouthernEthiopia [25], Gondar, Ethiopia [23] and Telangana, India [3] stated that the use of packed salt has positive association with increased contents of iodine. Open market salt is more prone to evaporation and iodine by its nature is highly volatile.

This study revealed that the odds of having adequate iodine concentration by households who did not expose for sunlight were about three times higher than the odds of iodized salt used by households who exposed it for sunlight. Studies from Gondar, Ethiopia [23], India [13] and London confirmed that heat can contribute the highest share for iodine loss. Sunlight exposure hastens the oxidation of iodide to elemental iodine and this is highly unstable.

The percentage of adequately iodized salt (68.8%) is not a warranty to conclude that utilization of iodine is sufficient unless we determine urinary iodine concentration of households in the study area. Due to the relatively small sample size and the winter season of data collection period, the finding of the study may not strong enough to represent the general population.

## Conclusions

In conclusion, availability of adequately iodized salt in Dessie and Combolcha towns was 68.8%. As compared to the targets of WHO recommendation and the Ethiopian- Federal Ministry of Health, the progress in the study area remaines slow. Only 25% of the households knew the benefit of iodized salt and most of them did not know negative effect of sunlight exposure.

The level of humidity, socio-economic status, use of iodized salt which is packed and did not expose to sunlight had significant association with adequacy of iodized salt. Working at grass root level is vital to achieve the desired target; to internalize the consequences of iodine deficiencies and proper utilization of iodized salt by enhancing the knowledge of individuals about the role of iodine and benefit of iodized salt. There should be periodical monitoring and evaluation of iodine particularly in areas where the use of open salt is common. Educating the households regarding proper practices of iodized salt use should be extensively addressed like not to expose for heat, adding salt at the end of cooking, placing in dry place and proper storage of salt in the house. There should be also regular and strick monitoring schedule at household level to take an immediate action.

## Abbreviations

ICCIDD: International Consultancy Committee for Iodine Deficiency Disorder
IDD: Iodine Deficiency Disorder
ODK: Open Data Kit
OR: Odds Ratio
PPM: Parts Per million
RTK: Rapid Test Kit
UIC: Urinary Iodine Concentration
USI: Universal Salt Iodization
